# Comparison of the Safety and Efficacy of Oral L-arginine for the Treatment of Cognitive Impairment in Older Adults: A Prospective, Randomized, Placebo-Controlled Pilot Study

**DOI:** 10.7759/cureus.112987

**Published:** 2026-07-20

**Authors:** Sonia Teotia, Abhay K Rana, Vani Gupta

**Affiliations:** 1 Physiology, Hind Institute of Medical Sciences, Barabanki, Barabanki, IND; 2 Anesthesiology and Critical Care, Max Super Speciality Hospital, Lucknow, Lucknow, IND; 3 Physiology, King George's Medical University, Lucknow, IND

**Keywords:** ace-iii, cognitive impairment, elderly, l-arginine, mci, mild cognitive impairment, randomized controlled trial

## Abstract

Background

Cognitive impairment (mild cognitive impairment and early dementia) is a growing concern with limited therapeutic options. L-arginine, a semi-essential amino acid and precursor of nitric oxide, has shown neuroprotective effects in animal studies, but human trials, particularly in India, are lacking. This pilot study aimed to assess the safety and efficacy of oral L-arginine in elderly patients with cognitive impairment.

Methodology

This was a prospective, double-blind, randomized, placebo-controlled pilot study conducted at a tertiary care hospital in North India. Sixty-six subjects aged ≥60 years with subjective cognitive complaints were screened using the Hindi version of Addenbrooke's Cognitive Examination III (ACE-III); those scoring ≤82 were included after excluding reversible causes, including low vitamin B12 levels and abnormal thyroid-stimulating hormone (TSH) levels, coronary artery disease (CAD), and substance use. Thirty-eight participants were randomized to receive either oral L-arginine 6.4 g/day or a matching placebo for 30 days. Cognitive function was reassessed post-intervention using ACE-III. Adverse effects and tolerability were recorded.

Results

Thirty-five subjects completed the study (16 L-arginine and 19 placebo). Baseline characteristics were comparable between groups. After 30 days, the L-arginine group showed statistically significant within-group improvements in attention (p=0.018), memory (p<0.001), and language (p=0.017) domains, while the placebo group improved only in memory (p=0.003). Between-group comparison showed that significantly more patients in the L-arginine group achieved normal ACE-III scores (>82) compared to placebo (37.5% vs. 5.3%, p=0.032). Adverse effects leading to withdrawal in the L-arginine group included thyroid dysfunction (one participant), nausea/diarrhea (one participant), and bitter taste (one participant); no withdrawals occurred in the placebo group (p=0.353).

Conclusions

Oral L-arginine (6.4 g/day for 30 days) appears safe and improves attention, memory, and language domains in elderly patients with cognitive impairment. Larger, longer-term trials are warranted to confirm these findings and assess progression to dementia.

## Introduction

Cognitive impairment, encompassing mild cognitive impairment (MCI) and dementia, represents a growing public health challenge among the elderly worldwide. MCI is an intermediate state between normal cognitive aging and dementia, characterized by subjective cognitive complaints and objective deficits on neuropsychological testing without significant functional impairment [1]. The prevalence of MCI using expanded Mayo Clinic criteria averages 18.9%, with annual progression rates to dementia of 10-15% [2]. Early diagnosis is crucial as it allows timely interventions, legal and financial planning, and access to support programs [3].

Despite extensive research, pharmacological options to halt or reverse cognitive decline remain limited. This has led to growing interest in nutraceuticals and dietary supplements that may modulate neurobiological pathways involved in cognitive function. L‑arginine, a semi‑essential amino acid, is the obligate substrate for nitric oxide (NO) synthesis via nitric oxide synthase (NOS) [4]. NO acts as a neurotransmitter, regulates cerebral blood flow, and influences synaptic plasticity, long‑term potentiation (LTP), and neurogenesis - all processes critical for learning and memory [5].

Animal studies have demonstrated that L‑arginine supplementation improves memory and attenuates cognitive deficits in models of Alzheimer’s disease and lipopolysaccharide‑induced neuroinflammation [6,7]. However, human trials, particularly in India, are lacking. Most existing studies have focused on L‑homoarginine or polyamines rather than L‑arginine itself, and none have evaluated oral L‑arginine in patients with established cognitive impairment using a validated Hindi screening tool [8].

Therefore, this prospective, double‑blind, randomized, placebo‑controlled pilot study was designed to assess the safety and efficacy of oral L‑arginine (6.4 g/day for 30 days) in elderly subjects with cognitive impairment, using the Hindi version of Addenbrooke’s Cognitive Examination III (ACE‑III) as the primary outcome measure.

## Materials and methods

The study was a prospective, double‑blind, randomized, placebo‑controlled pilot trial. Consecutive subjects attending the outpatient department of Geriatric Mental Health, King George’s Medical University (KGMU), Lucknow, India, between January 2019 and August 2019 were screened for participation.

The inclusion criteria were age ≥60 years, either sex; Hindi literate (to enable administration of the Hindi version of ACE-III); and ACE-III score ≤82 (indicative of cognitive impairment) [9]. The exclusion criteria were age <60 years; serum thyroid-stimulating hormone (TSH) and/or vitamin B12 below the normal range (reversible causes of cognitive impairment); deranged liver or renal function tests; history of coronary artery disease; psychosomatic or neurological disorder; smoking or tobacco use in any form; substance or alcohol abuse; and excessive caffeine intake.

As a pilot study with no prior Indian data on oral L‑arginine in cognitive impairment, a convenience sample of 38 consecutive eligible subjects was recruited (allowing for ~20% dropout). No formal power calculation was performed. Study parameters included those related to primary efficacy, namely change in total and domain‑specific ACE‑III scores (attention, memory, fluency, language, visuospatial) from baseline to day 30, and safety, namely adverse effects (gastrointestinal, thyroid dysfunction, taste disturbance) and drug discontinuation rates.

The study was approved by the Institutional Ethics Committee of KGMU, Lucknow (93rd ECM II B-Thesis/P45). Written informed consent was obtained from each participant or their legally acceptable representative. The study followed the ethical guidelines of the Declaration of Helsinki for human research.

Collection of demographic and baseline data was done using a detailed case sheet that recorded age, sex, education, occupation, marital status, duration of forgetfulness, medical history, and medication use. Fasting venous blood (3 mL) was drawn for serum TSH and vitamin B12 estimation using a fully automatic analyzer (VITROS 350 Chemistry System; Ortho-Clinical Diagnostics Inc., Rochester, NY, USA). Subjects with abnormal TSH/B12 were excluded.

Estimation of cognitive status was done using the Hindi version of ACE‑III [9]. ACE‑III is a 100‑point test covering five domains: attention (18 points), memory (26 points), fluency (14 points), language (26 points), and visuospatial (16 points). Administration time is 15-20 minutes. A cutoff of ≤82 was taken to define cognitive impairment, based on validation studies showing a sensitivity of 93% and a specificity of 100% for this cutoff [10]. The Hindi version has been standardized and used successfully in Indian populations.

A random character table was generated in Microsoft Excel (Microsoft Corp., Redmond, WA, USA) using the formula = CHAR(RANDBETWEEN(68,69)), which randomly assigned codes “D” or “E” to serial numbers 1 to 38. The codes corresponded to oral L‑arginine or placebo (roasted semolina powder), with the allocation key known only to the chief supervisor. Jars containing 120 capsules each (800 mg powder per capsule) were labeled with the code (e.g., 1D, 2E). Eligible subjects received jars according to the randomization table.

Subjects in the intervention group received oral L‑arginine powder (Nutrija Lifesciences, Nagda, MP, India) 800 mg per capsule. The daily dose was 6.4 g (eight capsules), taken as 2‑3‑3 capsules before each of three meals, for 30 days. The placebo group received identical capsules containing roasted semolina powder.

Compliance was ensured by regular telephone reminders and by checking returned empty jars at the end of 30 days. Subjects were asked to report any adverse effects (nausea, diarrhea, taste disturbance, or any new symptom) immediately. At the day‑30 visit, a structured enquiry was made for adverse events. After completion of the 30‑day intervention, all subjects underwent a repeat ACE‑III assessment by the same investigator, who remained blinded to group allocation.

Data were entered into Microsoft Excel and analyzed using IBM SPSS Statistics for Windows, Version 21 (Released 2012; IBM Corp., Armonk, New York, United States). Continuous variables were expressed as mean ± SD and compared using independent Student’s t‑test (between groups) or paired t‑test (within groups). Categorical variables were compared using the chi‑square test or Fisher’s exact test, as appropriate. A p-value <0.05 was considered statistically significant.

## Results

**Figure 1:**
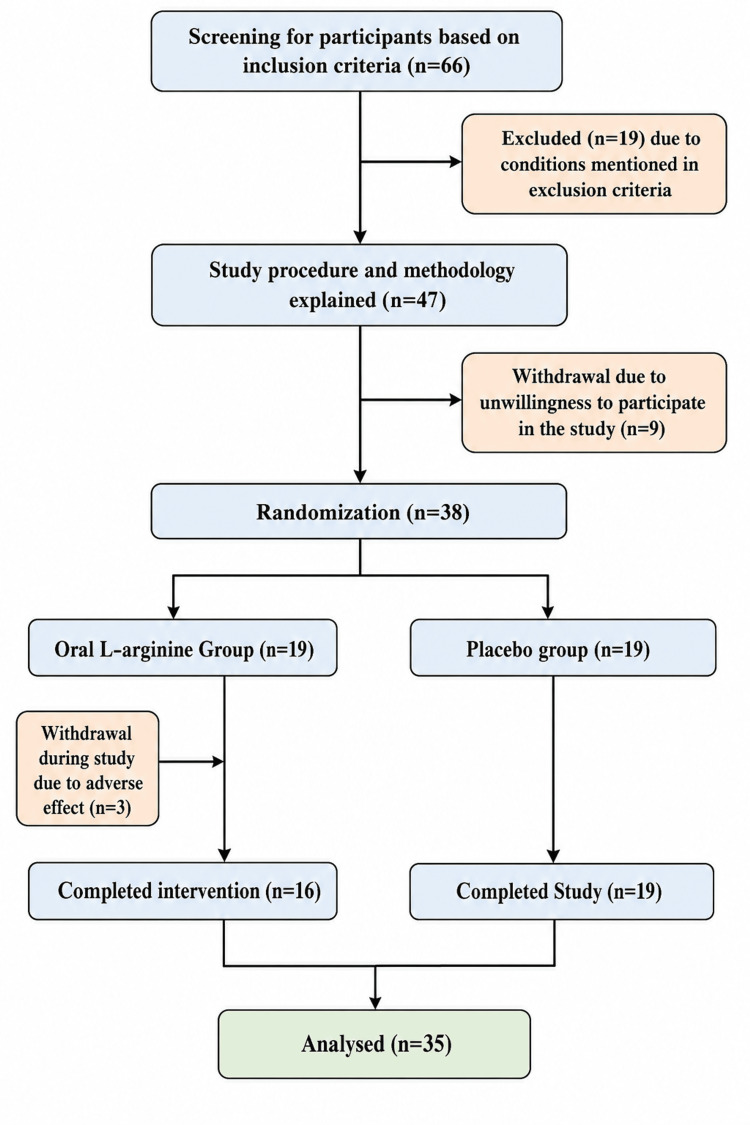
Consolidated Standards of Reporting Trials (CONSORT) flow diagram

A total of 66 subjects were screened; 19 were excluded due to exclusion criteria (mainly abnormal TSH/B12 or comorbid illnesses), and nine refused participation. Thus, 38 subjects were randomized. Three subjects in the L‑arginine group withdrew due to adverse effects (thyroid dysfunction, nausea/diarrhea, bitter taste), leaving 16 in the intervention group and 19 in the placebo group for final analysis (Figure 1).

Table 1 presents baseline characteristics. Both groups were comparable (p>0.05 for all). Mean age was 70.38±5.29 years in the L‑arginine group vs. 68.95±5.47 years in the placebo group. Males predominated (62.5% vs. 78.9%). Educational status was similar, with the majority being undergraduates. Mean duration of forgetfulness was 2.08±1.54 years in the intervention group vs. 1.66±1.11 years in the placebo group. Baseline serum TSH and vitamin B12 levels did not differ significantly. Baseline ACE‑III total scores were 55.13±21.46 (intervention) and 56.11±18.84 (placebo), with no significant differences in any of the five cognitive domains.

**Table 1:** Comparison of demographic profile and baseline characteristics of patients between the two study groups TSH: thyroid-stimulating hormone; ACE: Addenbrooke's Cognitive Examination

S. No.	Characteristic	Intervention Group (n=16)	Placebo Group (n=19)	p-value
1	Mean age±SD (range) years	70.38±5.29 (60-80)	68.95±5.47 (60-80)	0.441
2	Male: Female	10 (62.5%):6 (37.5%)	15 (78.9%):4 (21.1%)	0.283
3	Education			
	Primary/middle	4 (25.0%)	6 (31.6%)	0.497
	High school	5 (31.3%)	2 (10.5%)	
	Intermediate	1 (6.3%)	2 (10.5%)	
	Graduate and above	6 (37.5%)	9 (47.5%)	
4	Mean time since onset of disease±SD (range) years	2.08±1.54	1.66±1.11	0.357
		(1 month-6 years)	(7 days-4 years)	
5	Mean serum TSH±SD (range) (mIU/L)	1.92±1.02	2.36±1.07	0.231
		(0.67-3.84)	(1.07-4.36)	
6	Cognitive scores (ACE)	Mean±SD	Mean±SD	
	Attention	12.25±4.66	12.68±4.76	0.788
	Memory	11.06±6.72	10.79±5.08	0.892
	Fluency	4.06±2.43	4.26±1.76	0.779
	Language	18.38±5.45	18.84±5.80	0.809
	Visuospatial	9.38±4.15	9.53±0.99	0.917
	Total (range)	55.13±21.46	56.11±18.84	0.886
		20-82	14-79	

Table 2 shows post‑intervention ACE‑III scores. Although mean scores for attention, memory, language, and total were numerically higher in the L‑arginine group, between‑group differences were not statistically significant (p>0.05). However, the proportion of patients achieving normal ACE‑III scores (>82) after 30 days was significantly higher in the L‑arginine group (6/16, 37.5%) compared to placebo (1/19, 5.3%), with p=0.032 by Fisher’s exact test.

**Table 2:** Comparison of cognitive scores and outcomes between the two study groups at follow-up *p<0.05, **p<0.01, ***p<0.001 as compared to baseline. ACE: Addenbrooke's Cognitive Examination

S. No.	Characteristic	Intervention Group (n=16)	Placebo Group (n=19)	p-value
1	Cognitive scores (ACE)	Mean±SD	Mean±SD	-
	Attention	13.06±4.78*	12.84±5.00	0.895
	Memory	15.06±6.55***	13.47±6.50**	0.478
	Fluency	4.94±2.38	3.95±1.96	0.186
	Language	20.31±5.19*	18.16±4.68	0.206
	Visuospatial	9.94±3.82	9.11±4.38	0.557
	Total (range)	63.31±21.24	57.53±20.49	0.419
		(27-86)	(14-89)	
2	Number of patients achieving ACE-III scores >82	6 (37.5%)	1 (5.3%)	0.032
				Fisher's exact test

Table 3 summarizes adverse effects leading to drug withdrawal. In the L‑arginine group, one subject each developed thyroid dysfunction (TSH>4.5 mIU/L), nausea with diarrhea, and bitter taste, leading to discontinuation. No subject in the placebo group withdrew due to adverse effects. The overall difference in adverse event profile between groups was not statistically significant (χ^2^=3.257, df=3; p=0.353).

**Table 3:** Comparison of adverse effects leading to drug withdrawal between the two study groups TSH: thyroid-stimulating hormone

S. No.	Characteristic	Intervention Group (n=19)	Placebo Group (n=19)
1	Thyroid dysfunction (TSH>4.5 mIU/L)	1 (5.3%)	0
2	Nausea and diarrhea	1 (5.3%)	0
3	Discontinuation due to bitter taste	1 (5.3%)	0
4	No adverse effects	16 (84.2%)	19 (100%)

## Discussion

To the best of our knowledge, this is the first Indian randomized controlled trial to evaluate oral L‑arginine in elderly patients with cognitive impairment. After 30 days of supplementation (6.4 g/day), the L‑arginine group showed significant within‑group improvements in attention, memory, and language domains, whereas the placebo group improved only in memory. More importantly, significantly more patients in the L‑arginine group (37.5%) crossed the normal ACE‑III threshold (>82) compared to placebo (5.3%), suggesting a clinically meaningful benefit.

The memory improvement in the placebo group (24.88% change) may reflect a practice effect or spontaneous fluctuation, but the L‑arginine group showed a larger gain (36.17% change, p<0.001). The lack of significant between‑group differences in mean post‑intervention scores is likely due to the small sample size and high baseline variability.

Our findings align with animal studies showing that L‑arginine enhances memory via the NO-cyclic guanosine monophosphate (cGMP) pathway and promotes hippocampal neurogenesis [6,7]. L‑arginine is converted by neuronal NOS to NO, which acts as a retrograde messenger facilitating LTP [5]. Additionally, L‑arginine metabolites such as polyamines (putrescine, spermidine) have been shown to stimulate neural progenitor proliferation and differentiation [11].

The observed improvement in attention and language domains may be related to enhanced cerebral blood flow via endothelial NOS‑mediated vasodilation [12], as well as reduced oxidative stress and neuroinflammation [13]. The dual role of NO - neuroprotective at low concentrations but neurotoxic at high concentrations - is well recognized; our dose of 6.4 g/day appears to have been safe and within the therapeutic window. A protective effect of L-arginine on Alzheimer’s disease has also been shown in a recent experimental animal study by modulating hippocampal NO levels and memory deficits in aluminium chloride-induced rat models [14,15].

Adverse effects were mild and led to withdrawal in only 3/19 (15.8%) L‑arginine recipients, with no serious events. This safety profile is consistent with previous L‑arginine trials [16]. A recent meta-analysis evaluating the safety assessment of L-Arg oral intake recorded a no-observed adverse effect level (NOAEL) of 7531 mg/one-time dose. In this meta-analysis, an increase in the risk of gastrointestinal symptoms was found to be only 0.01 (95% CI 0.02-0.04) [17]. In our study, the adverse effects noted may not be solely attributable to L-Arg; however, the drug was withdrawn with patient interest as the first priority, and these withdrawals were made only for mild adverse effects.

Limitations

The present study had a small sample size (only 35 patients; 16 in the intervention group and 19 in the placebo group completed the assessment). The intervention period was of a short duration (30 days). We did not carry out any biomarker assessment (e.g., plasma arginine levels) to assess the possible mechanism of action. Further, there was an absence of long‑term follow‑up to assess progression to dementia.

## Conclusions

Oral L‑arginine (6.4 g/day for 30 days) appears safe and improves attention, memory, and language domains in elderly patients with cognitive impairment. A significantly higher proportion of L‑arginine‑treated patients achieved normal cognitive scores compared to placebo. These pilot findings warrant larger, longer‑term randomized controlled trials to confirm efficacy, explore dose‑response relationships, and evaluate whether L‑arginine can slow the progression from MCI to dementia.
